# On the Free Energy That Drove Primordial Anabolism

**DOI:** 10.3390/ijms10041853

**Published:** 2009-04-22

**Authors:** Michael Kaufmann

**Affiliations:** The Protein Chemistry Group, Witten/Herdecke University, Stockumer Str. 10, 58448 Witten, Germany; E-Mail: mika@uni-wh.de; Tel. +49-2302-926-387; Fax: +49-2302-926-220

**Keywords:** Primordial Anabolism, Origin of Life, Free Energy

## Abstract

A key problem in understanding the origin of life is to explain the mechanism(s) that led to the spontaneous assembly of molecular building blocks that ultimately resulted in the appearance of macromolecular structures as they are known in modern biochemistry today. An indispensable thermodynamic prerequisite for such a primordial anabolism is the mechanistic coupling to processes that supplied the free energy required. Here I review different sources of free energy and discuss the potential of each form having been involved in the very first anabolic reactions that were fundamental to increase molecular complexity and thus were essential for life.

## 1. Introduction

How and where did life on Earth arise [1]? This is one of the top 25 unanswered big questions facing science that were selected in the 125th anniversary issue of Science in 2005 [2]. Moreover, in the first essay of a series celebrating the 150th anniversary of Charles Darwin’s famous publication “*On the Origin of Species*” [3], Zimmer termed it one of the biggest questions in all of biology [4]. The quest for a better understanding of the processes involved in the origin of life naturally depends on the ability to discriminate living from non-living things. Scientifically spoken, a generally accepted definition of life would be very desirable for categorizing observed phenomena in being alive or not. Unfortunately, at present not even this initial goal has entirely been attained [5], let alone all approaches aimed at explaining the enigmatic transition of inanimate matter to living structures. In my opinion, the most successful endeavor to define life is the NASA working definition originally put by Joyce which reads: “Life is a self-sustained chemical system capable of undergoing Darwinian evolution” [6,7]. Szostak goes even a step further by stating that the origin of life and the origin of Darwinian evolution are essentially the same thing [4]. How difficult it is to define life can be seen easily by the substantially large number of further efforts to do so. On the basis of the compiled definitions and discussions during the 2001 meeting of the International Society for the History, Philosophy, and Social Studies of Biology, the following definition of life was composed: “Life is a succession of energy-producing electro-chemical processes by a naturally occurring, simple or complex organism composed of a combination of molecules, each consisting of systematically arranged carbon, hydrogen, and oxygen atoms, and a few other elements, forming cells, which consume ‘food’ and produce ‘waste’, both consisting of solid, aqueous, and gaseous matter; the process is called metabolism; the organism is capable of living within the environment without dependency on any other organism; energy use is manifest by growth with size limits for most; self-healing; possibly movement; self-replication with each offspring slightly different; irritability; capable of modifying their living environment, both beneficially and detrimentally; with eventual termination of energy production, or death. Exceptions are egg, sperm, spore, seed, and virus, which do not consume food and produce waste; the first four are replication structures, and the fifth has premature life-terminating capabilities” [8]. For a definition this is an enormously long phrase and in order to avoid counter-examples, this definition introduces exceptions from the rules it describes. In fact, the existence of counter-examples is a problem common to almost all attempts to define life. For instance, properties such as movement, growth, replication or Darwinian evolution are often part of definitions of life. Yet a cloud is able to move, a cactus is not. An avalanche is growing, elder humans do not. A crystal or a fire may replicate, a worker ant or bee does not. Even the property to evolve is not specific to life since certain macromolecules are capable of undergoing Darwinian evolution as well [9]. Two vintage quotations related to the question “What is life?” impressively demonstrate the dilemma mentioned. In the beginning of chapter 14 of his book “*What is Life*” Haldane writes: “I am not going to answer this question. In fact, I doubt if it will ever be possible to give a full answer” [10]. Similarly, Eigen recognizes the difficulty by stating: “Not only is this a difficult question; perhaps it is not even the right question” [11]. Indeed, more recently Cleland and Chyba argue that the controversy on life’s definition is inescapably as long as we lack a general theory of the nature of living systems [5]. Even worse, Schulze-Makuch and Irvin take into consideration that any rigid distinction between life and non-life is a matter of subjective judgment [12]. Koshland circumvents the problem by describing fundamental pillars on which life is based, essential principles by which a living system operates. In his essay “*The Seven Pillars of Life*” he summarizes them by the terms program, improvisation, compartmentalization, energy, regeneration, adaptability, and seclusion (abbreviated as PICERAS) [13]. To my mind, with respect to the origin of life energy is the most important pillar of his brilliant essay and consequently biological energy conversion plays a major role in life.

In 1944 Schrödinger published his famous book “*What is Life*”, in which he points out that life is avoiding the rapid decay into the inert state of equilibrium and that it is continually drawing negative entropy from its environment [14]. Although the term negative entropy or negentropy [15] created some confusion and his description is far from being a definition of life, Schrödinger already recognizes a very important basic principle that, with only a few exceptions, is unique to all things that are alive. Life is capable to create order and complexity, the opposite of entropy, by feeding on a suitable kind of free energy. In terms of biochemistry, the continuous supply of free energy is essential for anabolism, which is defined as the processes in metabolism that result in the synthesis of cellular components from precursors of low molecular weight [16]. Reactions that build up complex molecules from smaller building blocks are fundamental to every living being. As a consequence, the utilization of free energy, vitally important for every anabolic reaction, is essential for the origin of life as well.

In this contribution I strictly differentiate between the terms “primordial chemistry” and “primordial anabolism” as two consecutive achievements in the course of prebiotic evolution. I refer to primordial chemistry as the reactions leading to the synthesis of molecular building blocks as it was demonstrated for the first time by the Miller-Urey-Experiment, where amino acids were synthesized from less complex gaseous molecules that were believed to constitute early Earth’s atmosphere [17]. Unlike primordial chemistry as the chemical pathways resulting in small and simple molecular building blocks, I define primordial anabolism as the processes involved in the consecutive attachment of those building blocks resulting in a more complex and much larger macromolecular structure. As it will turn out, there is a fundamental difference between those two concepts. Primordial chemistry

- takes place spontaneously provided the presence of precursors and sufficient energy,
- is not hampered by energies high enough to be destructive for biological macromolecules and
- does not require any coupling to a suitable source of free energy.

The reaction products with comparably low molecular weight of primordial chemistry are certainly more robust than the macromolecules produced by primordial anabolism. The Miller-Urey-like synthesis of building blocks seems to occur without any guidance as if it was an intrinsic property of matter itself comparable with charge or mass. The physical properties of the known atoms and the laws of chemical bonding governing the building of molecules seem to imply that among others biochemical building blocks are favored compounds that form autonomously provided a sufficient amount of energy is available. On the contrary, all this does not hold true for primordial anabolism which is already somewhat advanced and more fragile. In general, anabolism depends on a coordinated coupling to a required form of free energy which in contemporary biochemistry is assured by high energy compounds such as ATP or other nucleosidetriphosphates and the substrate specificity of enzymes catalyzing anabolic polymerization reactions. The existence of primordial anabolism is also a prerequisite for information to come into play. Biological macromolecules are capable of storing information in the form of the sequential order of their building blocks, an important requirement for inheritance and thus Darwinian evolution as well [3]. Primordial anabolism was certainly an important step during the origin of life. Perhaps the emergence of primordial anabolism in the described sense and the origin of life were even identical events.

## 2. Energy

The etymological source of the word energy is the Greek word energeia (ɛ̓νɛ́ργɛια) which goes back to the philosophy of Aristotle [18] and means activity or being at work. Everybody intuitively has an idea about the nature of energy. Energy seems to be the entity needed to make things happen. It seems to be a kind of cause for the events that occur, an ingredient that brings about everything we can observe. Although discussed controversially [19], the classic textbook definition of energy in the field of physics is “the ability to do work” [20,21]. Energy has the same unit of measurement as work. Both physical quantities can be measured by the SI unit joule which, expressed in SI base units, corresponds to kg·m^2^·s^−2^. Richard Feynman stated: “There is a fact, or if you wish, a law governing all natural phenomena that are known to date. There is no known exception to this law – it is exact so far as we know. The law is called the conservation of energy” [22]. Energy obeys the fundamental physical law of conservation. In an isolated or closed system, energy can neither be created nor destroyed [23]. The first law of thermodynamics is an expression of this more universal physical law. Energy exists in several different forms. Although energy can be transformed or converted from one form into another one, in a closed system the total amount of energy always remains the same.

Thermodynamics describes the total internal energy of a system by the sum of “useless energy” and “useful energy”. In this context, “thermodynamic free energy” or in short “free energy” is the amount of internal energy that can be extracted from a system to do work. There is always “useless energy”, energy that cannot be extracted for work and which is lost in the form of heat. Two definitions of free energy, Gibbs free energy and Helmholtz free energy, are commonly in use. Whereas Gibbs free energy is defined under the assumption of constant pressure p and constant temperature T, Helmholtz free energy takes also into account the work done by p *d*V. Physically they are related by the following equations:

$$\begin{array}{c} G\;=\;U\;-\;TS\;+\;PV\\ F\;=\;U\;-\;TS\\ H\;=\;U\;+\;PV \end{array}$$

where *G* is the Gibbs free energy, *U* is the internal energy, *T* is the absolute temperature, *S* is the entropy, *P* is the absolute pressure, *V* is the volume, *F* is the Helmholtz free energy, and *H* is the enthalpy [24].

Free energy is the amount of energy that can be used to do work. Consequently, as an energy consuming process, anabolism depends on free energy as its driving force. Anabolism can be understood as a process of energy conversion where free energy is used to assemble preexisting molecular building blocks resulting in both the energy of newly formed high energy bonds and an increase in complexity and order, the latter being physically equivalent to the decrease of the system’s entropy [25]. Lehninger describes this essential feature of living organisms in his biochemistry textbook as “systems for extracting, transforming, and using energy from the environment, enabling organisms to build and maintain their intricate structures and to do mechanical, chemical, osmotic, and electrical work. This counteracts the tendency of all matter to decay toward a more disordered state, to come to equilibrium with its surroundings” [26]. Although some authors feel it is erroneous to refer to Schrödinger’s negentropy mentioned above as free energy, Schrödinger himself explains in a footnote of “*What is Life*” that by negative entropy he indeed means free energy [27]. The following paragraphs deal with different forms of energy, their respective roles in present day biochemistry as well as their potential to drive prebiotic chemistry eventually leading to the origin of life. Nuclear, atomic, gravitational, and relativistic energy are never discussed in the context of biological energy conversion and thus are also not included in this contribution as candidate energy sources for primordial anabolism.

## 3. Mechanical Energy

As the name suggests, mechanical energy is an energy form from the field of classical mechanics. Mechanical energy can be divided in potential energy and kinetic energy. Relative to a frame of reference, potential energy is defined as a function of the position and kinetic energy as a function of the movement of a classical object having mass. Some textbooks also group all further forms of energy into those two categories of energy with different notions of length scale.

Classical mechanics play an important role in biology. For instance, mechanical forces and mechanical work are involved in biochemical processes. It is known that cells can sense mechanical forces by switching the conformation of specialized proteins involved in signal transduction [28]. Energy provided by ATP/GTP hydrolysis or ion gradients across membranes can be converted to mechanical work as has been shown by the rotation of the bacterial flagellum [29] and the rotation of ATP-synthase [30] or by the movement of the multitude of further biochemical motor proteins [31] such as myosin in muscle contraction [32]. In contrast, neither kinetic nor potential energy as defined in classical mechanics is utilized by any known biological system as an energy source, let alone for anabolism.

A recent theory on prebiotic evolution developed by Hansma and designated the mica hypothesis [33,34] assumes that “Life may have originated between mica sheets, which would have provided many many confined spaces with surprising similarities to cells” [34]. The author also describes an energy source capable of rearranging molecules and the bonds between them forming biomolecules. According to her theory, mechanical energy from the movement of mica sheets as a response to ocean currents and temperature changes could provide the energy for the processes she assignes to the field of mechanochemistry. If true, the mechanochemical reactions proposed by Hansma certainly produce biochemical building blocks, possibly they also include condensation reactions as a part of primordial anabolism.

The environmental conditions on Earth today are comparably calm and bio-friendly. However, during the early days about 3.9 billion years ago and about 700 million years after the planets of our solar system had formed things were very different. At that time a cataclysmic spike in the cratering rate occurred as can be deduced from observations of the moon today [35,36]. This event is referred to as late heavy bombardment or lunar cataclysm and was characterized by a peak in the lunar impact rate by comets and asteroids that was about 100 times heavier than anything immediately before or after [37]. It is obvious that during the late heavy bombardment the early Earth was also subjected to massive impacts. Huge amounts of kinetic energy were stored in the projectiles when they were on their way towards their target Earth. At a constant velocity, the kinetic energy *E* of an asteroid is defined as:

$$E\;=\;½\;mv^{2}$$

where *m* is the mass, and *v* is the velocity.

Due to the considerably high velocities and notable masses involved, those impacts are accompanied by the absorption of tremendous quantities of kinetic energy. Even today, although fortunately with a much lower probability, cosmic impacts can in principle transfer energies sufficient enough to result in a global catastrophe and therefore still represent a permanent and serious danger for humankind [38,39]. The most popular example of such an event is the asteroid impact 65 million years ago which is believed to be the cause of the mass extinction at the Cretaceous/Tertiary boundary that ultimately led to the dying out of the dinosaurs [40]. More recently, the collision of comet Shoemaker- Levy 9 on Jupiter in 1994 impressively demonstrated the persistent danger from cometary impacts even today [41]. Beyond all question, impacts are hostile events and, provided that life has already come into existence before that as some authors suppose [42–44], the late heavy bombardment is assumed being a sterilizing period wiping out all such life forms, at least on the surface of the early Earth [45,46].

Rather than powering any kind of primordial anabolism, the kinetic energy delivered by impacts on early Earth, especially during the late heavy bombardment, surely destroyed any complex macromolecule that had possibly been already generated during the origin of life. Besides those deleterious effects on macromolecules, the impact energies could also have been favorable for primordial chemistry to take place. There is evidence that this kind of energy triggered the origin of the building blocks themselves in a manner similar to the one demonstrated by the Miller-Urey- Experiment [17]. Already in 1963, Gilvarry and Hochstim proposed that besides the classical energy sources of Miller-Urey-Experiments UV-irradiation from the Sun and lightning discharges in the atmosphere, in addition, the kinetic energy of meteorites when transformed to other energy forms during an impact could significantly contribute to the synthesis of organics [47]. Chyba and Sagan later reviewed the sources of organic molecules as the starting material for the origin of life [48]. They concluded that organics were delivered by extraterrestrial objects, synthesized by energy sources such as UV-light or electrical discharges, but also at comparable amounts synthesized via the energy derived from impact shocks. During this process, the impactor’s kinetic energy is converted into atmospheric shock heating resulting in the Miller-Urey-like synthesis of organic molecules such as amino acids. This mechanism has by now also been proved by laboratory experiments [49,50]. Even for hypervelocity impacts into ice as they took place on Jupiter’s moon Europa, energy conversions resulting in organic synthesis have been shown experimentally [51,52].

Impacts were possibly additionally involved in the origin of life by creating cracks and subsequently hydrothermal systems that might have been excellent incubators for prebiotic chemistry [53 54]. When absorbed by the impact crater the kinetic energy leads to a thermal anomaly generating a hydrothermal system. Such an environment could have provided conditions for the *de novo* synthesis of a diversity of organic compounds.

## 4. Electromagnetic Radiation

Electromagnetic radiation consists of a propagating wave of electric and magnetic fields. It can be described by the electromagnetic wave equation derived from Maxwell’s equations [55]. Depending on the wavelength in ascending order, it can be classified into radio waves, microwaves, infrared radiation, visible light, ultraviolet radiation, X-rays, and gamma rays. In a vacuum, electromagnetic radiation, regardless of what kind, propagates at the same constant velocity c, the speed of light [55]. According to the wave-particle duality electromagnetic radiation carries quantized energy in the form of photons [56]. The energy E carried by a photon is proportional to the radiation’s frequency and is given by

E = hν

where h is Planck’s constant and ν the frequency of the radiation [57].

Light from the Sun in the visible spectrum is the main natural source of electromagnetic radiation on Earth and absorbing photons from the Sun’s radiation is the major primary resource of free energy for biological processes on Earth today. Since Earth’s atmosphere is not transparent for gamma rays and ultraviolet rays [58], visible light is the window of the electromagnetic radiation spectrum with the highest frequency and thus the highest energy of individual photons that can reach the Earth’s surface. Even on primitive Earth visible light at wavelengths between 400 and 800 nm provided 70 mW·cm^−2^ which was by far more energy than electromagnetic radiation of all other wavelengths could deliver [59], and light energy was presumably also the most abundant source of energy on the prebiotic Earth [60]. An organism’s ability to transform the energy of photons from the Sun into biochemical energy in form of ATP is called phototrophy. If concomitantly CO_2_ is reduced to form biomass this kind of phototrophy is termed photosynthesis [61]. The importance of photosynthesis to biology in general is impressively demonstrated by the notion “big bang of evolution” when its origin is mentioned [62]. The most abundant electron donor for photosynthesis is water and during this so called oxygenic photosynthesis the majority of atmospheric oxygen is produced [63]. In general, phototrophic organisms absorb photons of visible light from the Sun at many different wavelengths [64].

Even in the absence of sunlight photosynthesis seems to be possible by absorbing geothermal light [65]. Geothermal light peaks in the infrared and is the black body radiation emitted from the heated rocks of hydrothermal vents and from their surrounding hot water [66]. Radiation consisting of frequencies higher than visible light is also discussed to play a role as an energy source. In a process called radiogenic metabolism, ionizing radiation such as ^60^Co gamma rays are hypothesized to promote metabolic reactions [67,68]. In a recent report, melanin pigments were described to change their electronic properties and melanized fungal cells manifested increased growth after exposure to ^188^Re induced ionizing radiation. The authors assume that melanotic organisms may harness radioactive radiation for metabolic energy [69].

There is no common consensus on whether phototrophic processes were at work during the origin of life. Some authors assume that photosynthesis could have played a role in providing free energy for the fixation of nitrogen and CO_2_ already at the very beginning [59,70,71] and others state that the use of light energy to drive biological reactions is almost as old as life itself [72]. Phylogenetic studies date the origin of phototrophy between 3.2 and 3.6 billion years ago with the last common ancestor and thus the origin of life more than 4.1 billion years ago [73]. Oxygenic photosynthesis is reported to be about 2.5 billion years old [74–76] which is consistent with the early rise in atmospheric oxygen concentration about 2.3 billion years ago [77,78]. Although discussed controversially [79,80], also much older times for its origin are reported. Des Marais concludes that oxygenic photosynthesis arose earlier than 2.8 billion years ago [81] and Schopf deduces from microfossil evidence that it was even 3.5 billion years ago [82,83]. Whatsoever, evolution took in the range of billions of years for its invention. This dramatically demonstrates the difficulty and the complexity of this sophisticated mechanism. Although photosynthesis is the most important process of energy conversion in contemporary biochemistry, the molecules involved even in the most primitive phototrophy today are much too complex for having been involved in energy transduction during the early steps of life’s origin. Bacteriorhodopsin, with only 247 amino acid residues, is by far the most simple modern macromolecule capable of performing energy transformations fueled by photons [26,84]. However, even the probability of the spontaneous assembly of a protein consisting of only 247 amino acids during the beginning of life is almost zero let alone the fact that in addition pigments and a cell membrane are required for its proper function. If primitive phototrophic processes were at work to power the origin of life, those processes can only have been based on very simple isolated pigments they were certainly anoxygenic. Due to its complexity, phototrophy, at least in all embodiments presently known, was not suitable to provide a source of free energy for primordial anabolism.

## 5. Chemical Energy

In the course of certain chemical reactions, huge amounts of free energy can be released. This change of free energy Δ*G* is expressed by the Gibbs-Helmholtz equation:

ΔG = ΔH − TΔS

where *G* is the Gibbs free energy, *H* is the enthalpy, *T* is the absolute temperature, and *S* is the entropy.

This equation characterizes the two driving forces of chemical reactions, namely the tendency to achieve a more stable bonding state as expressed by Δ*H* and the tendency to achieve a higher degree of randomness as expressed by Δ*S*. Under standard conditions, the change in free energy is directly related to the equilibrium constant by:

$$\Delta G{}^{\circ}\;=\;-RT\;\text{ln}(K_{\text{eq}})$$

where Δ*G°* is the change in standard Gibbs free energy, *R* is the gas constant, *T* is the absolute temperature, and *K*_eq_ = [products]/[substrates] is the equilibrium constant.

Δ*G°* is a characteristic constant associated to a given chemical reaction. A notable negative Δ*G°* implies that the products contain much less free energy than the substrates and that under standard conditions the reaction proceeds forward. Such reactions are termed exergonic. The free energy released by an exergonic reaction may be used to perform work or enable an unfavorable reaction (Δ*G°* positive) to proceed. In contemporary metabolism catabolic pathways deliver chemical energy in the form of the high energy compounds, mainly ATP, NADPH and NADH, which are used in anabolic pathways to convert small precursors into macromolecules [26]. A prerequisite for such processes is the coupling of two reactions, one delivering the free energy and a second one directly or indirectly involved in anabolism. For instance, the hydrolysis of ATP is accompanied by the release of free energy that is only useful for anabolism if the reaction is coupled to an anabolic one like the synthesis of aminoacyl-tRNAs in order to form proteins from amino acids or the synthesis of desoxynucleosidetriphosphates for the polymerization of DNA. In general, the use of chemical energy for anabolism is characterized by the coupling to reactions converting high energy compounds into molecules containing less free energy *i.e.* reactions characterized by a notably negative Δ*G°*.

Today the main high energy compounds that fuel catabolism obtain their energy directly or indirectly from photosynthesis [62]. In aerobic respiration these compounds release their free energy via oxidation by heterotrophic organisms and molecular oxygen as the electron acceptor is reduced to water [85]. Alternatively, the photosynthetic energy stored in the nutrients can be utilized anaerobically by fermentation, a process where instead of oxygen an endogenous organic compound is used as the electron acceptor [86]. However, some microorganisms, especially extremophiles, are capable of using chemical energy independently from both photosynthesis and aerobic respiration. Such processes, where ATP is generated via the respiratory chain in the absence of oxygen, are termed anaerobic respiration [87]. Some examples are special kinds of sulfur respiration and denitrification where molecular hydrogen and reduced sulfur compounds serve as electron donors while CO_2_, oxidized sulfur compounds, and NO_3_^−^ serve as electron acceptors [88].

Two main theories have emerged for the origin and early evolution of life based on heterotrophic versus chemoautotrophic metabolisms [89]. According to the heterotrophic theory molecular building blocks such as amino acids were continuously produced by primordial chemistry resulting in an organic soup from which primordial anabolism and life arose [90]. As the source of free energy, simple fermentations *i.e.* oxidations of preexisting reduced organic compounds are suggested [91]. However, to assemble these building blocks condensation reactions are needed. In liquid water these reactions are counteracted by the water’s mass effect which tends to prevent the accumulation of polycondensation products or polycondensation agents due to hydrolysis [92]. In contrast to the heterotrophic theory, the autotrophic theory proposes processes involving only low molecular primitive compounds without the need of preexisting building blocks [93,94]. For the autotrophic origin of life an elegant model including a suitable energy source was developed. According to Wächtershäuser’s iron-sulfur world hypothesis the formation of pyrite from ferrous ions and H_2_S is assumed to power all reducing anabolic reactions [95]. In an autocatalytic manner, carbon-fixation takes place by redox pathways. Catalyzed by transition metals, the reactions are driven by the free energy of inorganic starting materials, more precisely by the chemical potential of non-equilibrium volcanic exhalations [96]. Iron-sulfur clusters are probably a relic of the iron-sulfur world [97]. They are found in all life forms and are common to the most ancient components of living matter [98]. Some aspects of the iron-sulfur world hypothesis have already been confirmed experimentally [99–102].

As a matter of fact, if chemical energy is considered to be the only source of free energy in the origin of life, a continuous delivery of high energy compounds is required to maintain primordial anabolism as a premise for life. Life is anything but equilibrium [14,103]. Many molecules are discussed to be candidate high energy compounds driving primordial anabolism and most of them still play a role in contemporary metabolism. Phosphate esters and anhydrides dominate the living world [104], and ATP is the most important energy currency in biochemistry today [105]. Studies dealing with pyrophosphate-dependent phosphofructokinases that contain a latent ATP-binding site as a form of a molecular relic indicate that ATP itself or a closely related nucleotide could also have been the original high energy compound in the primeval Earth [106]. Pyrophosphate is part of the ATP molecule and all other nucleotides. It contains the high energy phosphoanhydride bond characteristic for all nucleosidetriphosphates. Pyrophosphate is produced in all reactions where ATP is degraded to AMP and its concomitant hydrolysis catalyzed by pyrophosphatases yielding two inorganic phosphates considerably contributes to drive those reactions [107]. Even the ability of pyrophosphate to replace ATP as the energy currency today is described in plants [108,109]. Pyrophosphate can also be detected in minerals termed canaphites [110]. Consequently, pyrophosphate was hypothesized to be a candidate primordial high energy compound in a scenario described as the PPi world [111–113]. Further candidate high energy compounds are thioesters such as Acetyl-CoA which is a central molecule of present metabolism [114]. Almost all metabolites can be degraded to Acetyl-CoA, and Acetyl-CoA itself is the building block of many biochemical structures such as fatty acids, cholesterol, and certain amino acids. The energy content of the thioester bond is sufficient to drive anabolic reactions and thus may have been involved in primordial anabolism as well. This theory was developed by De Duve and is commonly known as the thioester world hypothesis [115]. Further high-energy compounds suitable as a donor of free energy for primordial anabolism are glycine or other aminoacids [116], glyceraldehyde [117] or acetyl phosphate [118]. Formaldehyde and hydrogen cyanide are relatively stable, and at the same time their reactivity is high [119]. Although not being particularly high energy compounds they are considered to be key reactants in simulations of prebiotic chemical pathways [60]. Formaldehyde is described to be a precursor of sugars [120], and both amino acids and nucleic acid bases can be built from concentrated solutions of hydrogen cyanide [121–124].

## 6. Electrical Energy

Electrical energy is a form of potential energy generated by charge separation. If an electric current flows through a conductor, the electric energy *E* released can be quantified by:

E = UIt

where *U* is the voltage, *I* is the current, and *t* is the time.

With regard to electric energy in living systems the electric eel *Electrophorus electricus*, capable of generating voltages up to 500 V and currents of 1 A, is probably the most prominent example [125]. In biology considerable differences in ion concentrations are generated at many lipid membrane systems. This is a way to store energy by both an electrical potential consisting of the difference in charge and a chemical potential consisting of the difference in ion concentration. At the inner mitochondrial membrane as well as the thylakoid membrane of chloroplasts protons are pumped across the membrane and thereby establishing a pH gradient. This proton motive force stores the energy generated by respiration or photosynthesis [126]. The proton motive force in turn drives ATP synthesis catalyzed by ATP-synthase via a proton current directed backwards [30,127]. Beside the proton motive force there are further crucial ion gradients localized at membrane structures. Calcium gradients at the cell membrane and the sarcoplasmic reticulum are essential for triggering muscle contraction by suddenly releasing calcium into the cytoplasm along a decline in concentration when depolarization of a muscle cell occurs [128]. In addition, calcium influx driven by a calcium gradient plays an important role in cell signaling and thus calcium is described to act as second messenger in many cell types [129]. In general, the intracellular potassium concentration is much higher than the one outside the cell whereas the opposite is true for the sodium concentration. This concentration gradient is maintained by the ATP driven Na/K-ATPase [130] and is central for many cellular processes such as action potentials in neurons [131] or secondary active transport *e. g.* of glucose in the intestine and kidneys [132].

Energy storage in the form of a proton gradient as present in modern mitochondria is also hypothesized in a prebiotic context, and experiments show that some of the energy released by the exergonic conversion of micelles to vesicles can be stored into a transmembrane pH gradient [133]. Electric energy in the form of lightning and coronal discharges is believed to have provided energy for prebiotic organic synthesis on the early Earth [134,135]. This kind of energy is one of the energy forms used in the classical Miller-Urey experiment [17] which was reproduced several times, even by Miller himself [136]. Recent reanalyses of vials of his experiments using state of the art technology demonstrate that Miller synthesized 22 amino acids under conditions combining steam with electrical energy which supports spark discharge synthesis of organics by lightning in a steam-rich volcanic eruption [137]. Without any doubt electric energy in the form of lightning was important in prebiotic chemistry. On the other hand, it destroys large macromolecular polymers rather than synthesizing them as is shown by the severe injuries that can be caused by lightning [138,139]. Thus, this kind of electric energy can not have acted as a driving force for primordial anabolism.

## 7. Thermal Energy

The thermal energy of a system is proportional to its temperature. It is the sum of the system’s sensible heat and latent heat. Thermal energy can be converted to external work. This is the working principle of a heat engine where heat is transferred from a hot source to a cold sink and thereby some of it is converted into work or free energy. Consequently, a prerequisite to gain free energy from thermal energy is the presence of a suitable temperature gradient.

According to most textbooks, thermal energy is not utilized by contemporary biochemistry as the following passage illustrates: “Differences in temperature often exist between the internal and external environments of cells; however, cells generally cannot harness these heat differentials to do work. Even in warm-blooded animals that have evolved a mechanism for thermoregulation, the kinetic energy of molecules is used chiefly to maintain constant organismic temperatures” [140]. On the other hand, there are reports where even in biology thermal energy is considered as the source of free energy. The possibility of “Carnot creatures” capable of thriving on thermal gradients was even described in Nature’s column Daedalus [141]. The Carnot cycle is the most efficient thermodynamic cycle to convert thermal energy to perform external work [142]. Specialized organisms must have evolved to use the rich source of energy provided by black smokers, the argument goes. Matsuno states that actomyosin functions as a heat engine that is able to maintain a constant velocity due to quantum mechanical coherence and entanglement [143]. Interestingly enough, during the 18^th^ century, before the true biochemistry of muscle contraction was settled, it was indeed assumed that the mechanism of muscle contraction was based on the working principle of a heat engine [144]. In a more recent paper, Matsuno stated that molecular organizations leading to the origin of the phenomenon of life might have been associated with the emergence of a quantum coherence embodied in a robust heat engine feeding on quantum decoherence [145].

If at all, thermal energy at most plays a minor role in energy conversion of current biology. Nevertheless, during the origin of life it could have been much more important or even essential. Muller proposes pF1, a progenitor of modern ATP-synthase, which is driven by thermal cycling by a process he designates as thermosynthesis [146–148]. Muller postulates a thermally-induced binding change mechanism for pF1 ATP-synthase. During a thermal cycle the enzyme loosely binds ADP and phosphate at a low temperature, folds, converts these substrates to tightly bound ATP, and releases this tightly bound ATP at a high temperature by unfolding. In principle, a molecule like pF1 can condense any two substrates *e.g*. two amino acids and thus by definition converts free energy for primordial anabolism. pF1 not necessarily has to be a large molecule. For its proper function, it is sufficient to bind its substrates and adopt open and closed conformations. The smallest enzyme occurring naturally, 4-oxalocrotonate tautomerase, consists of about 60 amino acids [149], and the hammerhead ribozyme as the smallest natural RNA catalyst consists of only 30 nucleotides [150]. Much smaller peptide or RNA catalysts have been constructed *in vitro* shrinking the size of the smallest peptide catalyst to 29 amino acids [151] and the size of the smallest ribozyme to only five nucleotides [152]. Even the single amino acid proline is described acting as an enzyme [153]. Hence, it is conceivable that the assembly of the proposed pF1 catalyst can have happened solely by chance events. Possible candidate pF1 molecules are the proteinoids experimentally discovered by Fox [154]. Interestingly, the synthesis of those proteinoids themselves from amino acids is achieved by heating and drying and thus is also driven by thermal energy. Muller did not only propose pF1 but also progenitors of the photosynthetic machinery. According to him these progenitors worked as heat engines by thermotropic phase transitions in an asymmetric biomembrane during thermal cycling [155]. With respect to thermosynthesis unfortunately no experimental approaches are currently described in literature.

## 8. Conclusions

The nature of the free energy sources that have driven the earliest anabolic reactions remain a matter of speculation. Whereas a coupling mechanism is not necessary for the generation of molecular building blocks in primordial chemistry as demonstrated in Miller-Urey experiments, there is the need to couple primordial anabolic and thus endergonic polymerization reactions to either exergonic reactions utilizing a high energy compound or somehow directly to a suitable source of free energy. In addition, all mechanisms involved in primordial anabolism must be extremely simple so that their origin can be solely explained by chance events having taken place with acceptable probabilities. Because of its high degree of complexity, energy conversion as realized in contemporary biochemistry can certainly be excluded from having been at work during the earliest days of life. Currently, the most simple and most efficient mechanism to drive primordial anabolism is the reaction pathway described by Wächtershäuser’s iron-sulfur world hypothesis. Although the efficiency of thermosynthesis as proposed by Muller is rather low, its relative simplicity makes it an attractive alternative candidate. Whereas a huge amount of experimental data with respect to primordial chemistry is available, the experimental work to shed light on primordial anabolism is still in its infancy and thus a challenge for future origin of life research.

## References

[1] Zimmer C (2005). How and where did life on Earth arise?. Science.

[2] Kennedy D, Norman C (2005). What don’t we know?. Science.

[3] Darwin C (1859). On the origin of species by means of natural selection, or the preservation of favoured races in the struggle for life.

[4] Zimmer C (2009). Evolutionary roots. On the origin of life on Earth. Science.

[5] Cleland CE, Chyba CF (2002). Defining ‘life’. Orig. Life Evo. Biosph.

[6] Joyce GF, Deamer DW, Fleischhacker GR (1994). Origins of Life: the Central Concepts.

[7] Joyce GF, Zuckerman B, Hart M (1994). The RNA World: Life before DNA and Protein. Extraterrestrials - Where Are they? II.

[8] Winder CG (2001). What is life? Define life. ISHPSSB 2001 Meeting of the International Socoety for the History, Philosophy, and Social Studies of Biology.

[9] Joyce GF (2007). Forty years of in vitro evolution. Angew. Chem. Int. Ed. Engl.

[10] Haldane JBS (1949). What is life.

[11] Eigen M, Murphy MP, O’Neill LAJ (1995). What will endure of 20th century biology?. What is life? The next fifty Years.

[12] Schulze Makuch D, Irvin LN (2004). Life in the Universe. Expectations and Constrains. Advances in Astrobiology and Biogeophysics.

[13] Koshland DE (2002). Special essay. The seven pillars of life. Science.

[14] Schrödinger E (1944). What is life: the physical aspect of the living cell.

[15] Brillouin L (1953). Negentropy Principle of Information. J. Appl. Phys.

[16] De Bolster WWG (1997). Glossary of terms used in bioinorganic chemistry. Pure Appl. Chem.

[17] Miller SL (1953). A production of amino acids under possible primitive earth conditions. Science.

[18] Blair GA (1992). Energeia and entelecheia: Act in Aristotle.

[19] Lehrman RL (1973). Energy is Not the Ability to do Work. Phys. Teach.

[20] Iona M (1973). Energy is the ability to do work. Phys. Teach.

[21] Hobson A (2006). Physics: Concepts and Connections.

[22] Feynman R (1964). The Feynman Lectures on Physics.

[23] Grove WR (1846). On The Correlation of Physical Forces.

[24] Schroeder DV (2000). An Indroduction to Thermal Physics.

[25] Sethna JP (2006). Statistical mechanics: Entropy, order parameters and complexity.

[26] Lehninger A, Nelson LN, Cox MM (2008). Principles of Biochemistry.

[27] Ho MW, Gnaiger E, Gellerich FN, Wyss M (1994). What is (Schrödinger’s) Negentropy?. What is Controlling Life? 50 years after Schrödinger’s What is Life?.

[28] Vogel V (2006). Mechanotransduction involving multimodular proteins: converting force into biochemical signals. Annu. Rev. Biophys. Biomol. Struct.

[29] Terashima H, Kojima S, Homma M (2008). Flagellar motility in bacteria structure and function of flagellar motor. Int. Rev. Cell Mol. Biol.

[30] Stock D, Gibbons C, Arechaga I, Leslie AG, Walker JE (2000). The rotary mechanism of ATP synthase. Curr. Opin. Struct. Biol.

[31] van den Heuvel MG, Dekker C (2007). Motor proteins at work for nanotechnology. Science.

[32] Smith DA, Geeves MA, Sleep J, Mijailovich SM (2008). Towards a unified theory of muscle contraction. I: foundations. Ann. Biomed. Eng.

[33] Hansma H Mica and the origin of life: Cells without membranes The American Society for Cell Biology 47th Annual MeetingOASIS; The Online Abstract Submission and Invitation System:Washington, DC, USA 2007

[34] Hansma H (2008). Mica, bioenergetics, and the origin of life. Biophys. J.

[35] Gomes R, Levison HF, Tsiganis K, Morbidelli A (2005). Origin of the cataclysmic Late Heavy Bombardment period of the terrestrial planets. Nature.

[36] Cohen BA, Swindle TD, Kring DA (2000). Support for the lunar cataclysm hypothesis from lunar meteorite impact melt ages. Science.

[37] Kerr RA (2000). PLANETARY SCIENCE: Beating Up on a Young Earth, and Possibly Life. Science.

[38] Morrison D (2006). Asteroid and comet impacts: the ultimate environmental catastrophe. Philos. Transact. A Math. Phys. Eng. Sci.

[39] Gritzner C, Durfeld K, Kasper J, Fasoulas S (2006). The asteroid and comet impact hazard: risk assessment and mitigation options. Naturwissenschaften.

[40] Alvarez LW, Alvarez W, Asaro F, Michel HV (1980). Extraterrestrial Cause for the Cretaceous- Tertiary Extinction. Science.

[41] Weaver HA, A’Hearn MF, Arpigny C, Boice DC, Feldman PD, Larson SM, Lamy P, Levy DH, Marsden BG, Meech KJ (1995). The Hubble Space Telescope (HST) observing campaign on comet Shoemaker-Levy 9. Science.

[42] Nemchin AA, Whitehouse MJ, Menneken M, Geisler T, Pidgeon RT, Wilde SA (2008). A light carbon reservoir recorded in zircon-hosted diamond from the Jack Hills. Nature.

[43] Mojzsis SJ, Arrhenius G, McKeegan KD, Harrison TM, Nutman AP, Friend CR (1996). Evidence for life on Earth before 3,800 million years ago. Nature.

[44] Wilde SA, Valley JW, Peck WH, Graham CM (2001). Evidence from detrital zircons for the existence of continental crust and oceans on the Earth 4.4 Gyr ago. Nature.

[45] Maher KA, Stevenson DJ (1988). Impact frustration of the origin of life. Nature.

[46] Chyba CF (1993). The violent environment of the origin of life: progress and uncertainties. Geochim. Cosmochim. Acta.

[47] Gilvarry JJ, Hochstim AR (1963). Possible role of meteroites in the origin of life. Nature.

[48] Chyba C, Sagan C (1992). Endogenous production, exogenous delivery and impact-shock synthesis of organic molecules: an inventory for the origins of life. Nature.

[49] McKay CP, Borucki WJ (1997). Organic synthesis in experimental impact shocks. Science.

[50] Miyakawa S, Murasawa K, Kobayashi K, Sawaoka AB (2000). Abiotic synthesis of guanine with high-temperature plasma. Orig. Life Evol. Biosph.

[51] Borucki JG, Khare B, Cruikshank DP (2002). A new energy source for organic synthesis in Europa’s surface ice. J. Geophys. Res.

[52] Nna-Mvondo D, Khare B, Ishihara T, McKay CP (2008). Experimental impact shock chemistry on planetary icy satellites. Icarus.

[53] Cockell CS, Osinski GR, Lee P (2003). The impact crater as a habitat: effects of impact processing of target materials. Astrobiology.

[54] Cockell CS (2006). The origin and emergence of life under impact bombardment. Philos. Trans. R. Soc. Lond. B Biol. Sci.

[55] Maxwell JC (1865). A dynamical theory of the electromagnetic field. Philos. Trans. R. Soc. Lon.

[56] Einstein A (1905). Über einen die Erzeugung und Verwandlung des Lichtes betreffenden heuristischen Gesichtspunkt. Ann. Phys.

[57] Planck M (1901). Über das Gesetz der Energieverteilung im Normalspectrum. Ann. Phys.

[58] Bahcall JN, Spitzer L (1982). The space telescope. Sci. Am.

[59] Mauzerall D (1992). Light, iron, Sam Granick and the origin of life. Photosynth. Res.

[60] Deamer DW (1997). The first living systems: a bioenergetic perspective. Microbiol. Mol. Biol. Rev.

[61] Bryant DA, Frigaard NU (2006). Prokaryotic photosynthesis and phototrophy illuminated. Trends. Microbiol.

[62] Barber J (2004). Engine of life and big bang of evolution: a personal perspective. Photosynth. Res.

[63] Barber J (2008). Photosynthetic generation of oxygen. Philos. Trans. R. Soc. Lond. B Biol. Sci.

[64] Kiang NY, Siefert J, Govindjee, Blankenship RE (2007). Spectral signatures of photosynthesis. I. Review of Earth organisms. Astrobiology.

[65] Beatty JT, Overmann J, Lince MT, Manske AK, Lang AS, Blankenship RE, Van Dover CL, Martinson TA, Plumley FG (2005). An obligately photosynthetic bacterial anaerobe from a deep-sea hydrothermal vent. Proc. Natl. Acad. Sci. USA.

[66] White SN, Chave AD, Reynolds GT, Van Dover CL (2002). Ambient light emission from hydrothermal vents on the mid-atlantic ridge. Geophys. Res. Lett.

[67] Benford MS (2001). Radiogenic metabolism: an alternative cellular energy source. Med. Hypotheses.

[68] Luckey TD (1980). Radiogenic metabolism. Am. J. Clin. Nutr.

[69] Dadachova E, Bryan RA, Huang X, Moadel T, Schweitzer AD, Aisen P, Nosanchuk JD, Casadevall A (2007). Ionizing radiation changes the electronic properties of melanin and enhances the growth of melanized fungi. PLoS ONE.

[70] Hartman H (1998). Photosynthesis and the origin of life. Orig. Life Evol. Biosph.

[71] Mauzerall DC (1990). The photochemical origins of life and photoreaction of ferrous ion in the archaean oceans. Orig. Life Evol. Biosph.

[72] Rutherford AW, Nitschke W, Baltscheffsky H (1996). Photosystem II and the quinone-iron-containing reaction centers: comparison and evolutionary perspectives. Origin and evolution of biological energy conversion.

[73] Battistuzzi FU, Feijao A, Hedges SB (2004). A genomic timescale of prokaryote evolution: insights into the origin of methanogenesis, phototrophy, and the colonization of land. BMC Evol. Biol.

[74] Summons RE, Jahnke LL, Hope JM, Logan GA (1999). 2-Methylhopanoids as biomarkers for cyanobacterial oxygenic photosynthesis. Nature.

[75] Xiong J (2006). Photosynthesis: what color was its origin?. Genome. Biol.

[76] Raymond J, Zhaxybayeva O, Gogarten JP, Gerdes SY, Blankenship RE (2002). Whole-genome analysis of photosynthetic prokaryotes. Science.

[77] Kasting JF (2001). Earth history. The rise of atmospheric oxygen. Science.

[78] Bekker A, Holland HD, Wang PL, Rumble D, Stein HJ, Hannah JL, Coetzee LL, Beukes NJ (2004). Dating the rise of atmospheric oxygen. Nature.

[79] Towe KM (2001). On the origins of photosynthesis. Science.

[80] Brasier MD, Green OR, Jephcoat AP, Kleppe AK, Van Kranendonk MJ, Lindsay JF, Steele A, Grassineau NV (2002). Questioning the evidence for Earth’s oldest fossils. Nature.

[81] De Marais DJ (2000). Evolution. When did photosynthesis emerge on Earth?. Science.

[82] Schopf JW (1993). Microfossils of the Early Archean Apex chert: new evidence of the antiquity of life. Science.

[83] Schopf JW, Packer BM (1987). Early Archean (3.3-billion to 3.5-billion-year-old) microfossils from Warrawoona Group, Australia. Science.

[84] Oesterhelt D, Stoeckenius W (1973). Functions of a new photoreceptor membrane. Proc. Natl. Acad. Sci. USA.

[85] Das J (2006). The role of mitochondrial respiration in physiological and evolutionary adaptation. Bioessays.

[86] Voet D, Voet J (2005). Biochemistry.

[87] Zannoni D (2004). Advances in photosynthesis and respiration.

[88] Stetter KO (2006). Hyperthermophiles in the history of life. Philos. Trans. R. Soc. Lond. B Biol. Sci.

[89] Ferry JG, House CH (2006). The stepwise evolution of early life driven by energy conservation. Mol. Biol. Evol.

[90] Lazcano A, Miller SL (1999). On the origin of metabolic pathways. J. Mol. Evol.

[91] Russell MJ, Martin W (2004). The rocky roots of the acetyl-CoA pathway. Trends Biochem. Sci.

[92] Wachtershauser G (2006). From volcanic origins of chemoautotrophic life to Bacteria, Archaea and Eukarya. Philos. Trans. R. Soc. Lond. B Biol. Sci.

[93] Wachtershauser G (1988). Before enzymes and templates: theory of surface metabolism. Microbiol. Rev.

[94] Hartman H (1975). Speculations on the origin and evolution of metabolism. J. Mol. Evol.

[95] Wachtershauser G (1993). The cradle chemistry of life: On the origin of natural products in a pyritepulled chemoautotrophic origin of life. Pure Appl. Chem.

[96] Wachtershauser G (2007). On the chemistry and evolution of the pioneer organism. Chem. Biodivers.

[97] Wachtershauser G (1990). Evolution of the first metabolic cycles. Proc. Natl. Acad. Sci. USA.

[98] Beinert H, Holm RH, Munck E (1997). Iron-sulfur clusters: nature’s modular, multipurpose structures. Science.

[99] Huber C, Wachtershauser G (1997). Activated acetic acid by carbon fixation on (Fe,Ni)S under primordial conditions. Science.

[100] Huber C, Wachtershauser G (2006). alpha-Hydroxy and alpha-amino acids under possible Hadean, volcanic origin-of-life conditions. Science.

[101] Huber C, Wachtershauser G (1998). Peptides by activation of amino acids with CO on (Ni,Fe)S surfaces: implications for the origin of life. Science.

[102] Huber C, Eisenreich W, Hecht S, Wachtershauser G (2003). A possible primordial peptide cycle. Science.

[103] Egolf DA (2002). Statistical mechanics. Far from equilibrium. Science.

[104] Westheimer FH (1987). Why nature chose phosphates. Science.

[105] Itoh H, Takahashi A, Adachi K, Noji H, Yasuda R, Yoshida M, Kinosita K (2004). Mechanically driven ATP synthesis by F1-ATPase. Nature.

[106] Chi A, Kemp RG (2000). The primordial high energy compound: ATP or inorganic pyrophosphate?. J. Biol. Chem.

[107] Peller L (1977). Thermodynamic limits on the size and size distribution of nucleic acids synthesized in vitro: the role of pyrophosphate hydrolysis. Biochemistry.

[108] Huang S, Colmer TD, Millar AH (2008). Does anoxia tolerance involve altering the energy currency towards PPi?. Trends Plant. Sci.

[109] Serrano A, Perez-Castineira JR, Baltscheffsky M, Baltscheffsky H (2007). H+-PPases: yesterday, today and tomorrow. IUBMB Life.

[110] Rouse RC, Peacor DR, Freed RL (1988). Pyrophosphate groups in the structure of canaphite, CaNa 2 P 2 O 7 .4H 2 O; the first occurrence of a condensed phosphate as a mineral. Amer. Mineral.

[111] Baltscheffsky H, Ponnaperuma C, Chela-Flores J (1993). Chemical origin and early evolution of biological energy conversion. Chemical evolution: Origin of Life.

[112] Baltscheffsky H (1997). Major “anastrophes” in the origin and early evolution of biological energy conversion. J. Theor. Biol.

[113] Baltscheffsky H, Blomberg C, Liljenstrom H, Lindahl BI, Arhem P (1997). On the origin and evolution of life: an introduction. J. Theor. Biol.

[114] Bender-Machado L, Bauerlein M, Carrari F, Schauer N, Lytovchenko A, Gibon Y, Kelly AA, Loureiro M, Muller-Rober B, Willmitzer L, Fernie AR (2004). Expression of a yeast acetyl CoA hydrolase in the mitochondrion of tobacco plants inhibits growth and restricts photosynthesis. Plant Mol. Biol.

[115] De Duve C (1991). Blueprint for a cell.

[116] Clarke PH, Elsden SR (1980). The earliest catabolic pathways. J. Mol. Evol.

[117] Weber AL (1987). The triose model: glyceraldehyde as a source of energy and monomers for prebiotic condensation reactions. Orig. Life Evol. Biosph.

[118] Martin W, Russell MJ (2007). On the origin of biochemistry at an alkaline hydrothermal vent. Philos. Trans. R. Soc. Lond. B Biol. Sci.

[119] Pavlovskaya TE, Telegina TA (1974). Photochemical conversions of lower aldehydes in aqueous solutions and in fog. Orig. Life.

[120] Butlerow A (1861). Formation synthetique d’une substance sucree. Compt. Rend. Acad. Sci.

[121] Oro J, Kamat SS (1961). Amino-acid synthesis from hydrogen cyanide under possible primitive earth conditions. Nature.

[122] Schwartz AW, Voet AB, Van der Veen M (1984). Recent progress in the prebiotic chemistry of HCN. Orig. Life.

[123] Ferris JP, Hagan WJ (1984). HCN and chemical evolution: the possible role of cyano compounds in prebiotic synthesis. Tetrahedron.

[124] Oro J (1961). Mechanism of synthesis of adenine from hydrogen cyanide under possible primitive earth conditions. Nature.

[125] Gotter AL, Kaetzel MA, Dedman JR (1998). Electrophorus electricus as a model system for the study of membrane excitability. Comp. Biochem. Physiol. A Mol. Integr. Physiol.

[126] Mitchell P (1961). Coupling of phosphorylation to electron and hydrogen transfer by a chemi-osmotic type of mechanism. Nature.

[127] Boyer PD (1998). ATP synthase–past and future. Biochim. Biophys. Acta.

[128] Berchtold MW, Brinkmeier H, Muntener M (2000). Calcium ion in skeletal muscle: its crucial role for muscle function, plasticity, and disease. Physiol. Rev.

[129] Vig M, Kinet JP (2009). Calcium signaling in immune cells. Nat. Immunol.

[130] Geering K (2008). Functional roles of Na,K-ATPase subunits. Curr. Opin. Nephrol. Hypertens.

[131] Bean BP (2007). The action potential in mammalian central neurons. Nat. Rev. Neurosci.

[132] Brown GK (2000). Glucose transporters: structure, function and consequences of deficiency. J. Inherit. Metab. Dis.

[133] Chen IA, Szostak JW (2004). Membrane growth can generate a transmembrane pH gradient in fatty acid vesicles. Proc. Natl. Acad. Sci. USA.

[134] Chyba C, Sagan C (1991). Electrical energy sources for organic synthesis on the early Earth. Orig. Life Evol. Biosph.

[135] Miller SL, Urey HC (1959). Organic compound synthesis on the primitive earth. Science.

[136] Ring D, Wolman Y, Friedmann N, Miller SL (1972). Prebiotic synthesis of hydrophobic and protein amino acids. Proc. Natl. Acad. Sci. USA.

[137] Johnson AP, Cleaves HJ, Dworkin JP, Glavin DP, Lazcano A, Bada JL (2008). The Miller volcanic spark discharge experiment. Science.

[138] Mistovich JJ, Krost WS, Limmer DD (2008). Beyond the basics: lightning-strike injuries. EMS Mag.

[139] Ritenour AE, Morton MJ, McManus JG, Barillo DJ, Cancio LC (2008). Lightning injury: a review. Burns.

[140] Lodish H, Berk A, Zipursky LS, Matsudaira P, Baltimore D, Darnell J (2000). Molecular cell biology.

[141] Jones D (1997). The dark is light enough. Nature.

[142] Carnot S (1824). Réflexions sur la puissance motrice du feu et sur les machines propres à développer cette puissance.

[143] Matsuno K (1999). Cell motility as an entangled quantum coherence. Biosystems.

[144] Needham DM (1971). Machina carnis The biochemistry of muscular contraction in its historical development.

[145] Matsuno K (2006). Forming and maintaining a heat engine for quantum biology. Biosystems.

[146] Muller AW, Schulze-Makuch D (2006). Thermal energy and the origin of life. Orig. Life Evol. Biosph.

[147] Muller AW (1995). Were the first organisms heat engines? A new model for biogenesis and the early evolution of biological energy conversion. Prog. Biophys. Mol. Biol.

[148] Muller AW (1996). Hypothesis: the thermosynthesis model for the origin of life and the emergence of regulation by Ca2+. Essays Biochem.

[149] Chen LH, Kenyon GL, Curtin F, Harayama S, Bembenek ME, Hajipour G, Whitman CP (1992). 4-Oxalocrotonate tautomerase, an enzyme composed of 62 amino acid residues per monomer. J. Biol. Chem.

[150] Wedekind JE, McKay DB (1998). Crystallographic structures of the hammerhead ribozyme: relationship to ribozyme folding and catalysis. Annu. Rev. Biophys. Biomol. Struct.

[151] Atassi MZ, Manshouri T (1993). Design of peptide enzymes (pepzymes): surface-simulation synthetic peptides that mimic the chymotrypsin and trypsin active sites exhibit the activity and specificity of the respective enzyme. Proc. Natl. Acad. Sci. USA.

[152] Bombard S, Kozelka J, Favre A, Chottard JC (1998). Probing the mechanism of an Mn^2+^- dependent ribozyme by means of platinum complexes. Eur. J Biochem.

[153] Movassaghi M, Jacobsen EN (2002). Chemistry. The simplest “enzyme”. Science.

[154] Fox SW (1956). Evolution of protein molecules and thermal synthesis of biochemical substances. Am. Sci.

[155] Muller A (1993). W.J. A mechanism for thermosynthesis based on a thermotropic phase transition in an asymmetric biomembrane. Physiol. Chem. Physics Med. NMR.

